# Curiosity Is Contagious: A Social Influence Intervention to Induce Curiosity

**DOI:** 10.1111/cogs.12937

**Published:** 2021-02-12

**Authors:** Rachit Dubey, Hermish Mehta, Tania Lombrozo

**Affiliations:** ^1^ Department of Computer Science Princeton University; ^2^ Department of Electrical Engineering and Computer Sciences University of California, Berkeley; ^3^ Department of Psychology Princeton University

**Keywords:** Curiosity, Exploration, Learning, Intervention, Social influence

## Abstract

Our actions and decisions are regularly influenced by the social environment around us. Can social cues be leveraged to induce curiosity and affect subsequent behavior? Across two experiments, we show that curiosity is contagious: The social environment can influence people's curiosity about the answers to scientific questions. Participants were presented with everyday questions about science from a popular on‐line forum, and these were shown with a high or low number of up‐votes as a social cue to popularity. Participants indicated their curiosity about the answers, and they were given an opportunity to reveal a subset of those answers. Participants reported greater curiosity about the answers to questions when the questions were presented with a high (vs. low) number of up‐votes, and they were also more likely to choose to reveal the answers to questions with a high (vs. low) number of up‐votes. These effects were partially mediated by surprise and by the inferred usefulness of knowledge, with a more dramatic effect of low up‐votes in reducing curiosity than of high up‐votes in boosting curiosity. Taken together, these results highlight the important role social information plays in shaping our curiosity.

## 1 Introduction

Education takes for granted that sight is there but that it isn't turned the right way or looking where it ought to look, and tries to redirect it accordingly.Plato, *Republic*

From Sophocles's *Oedipus* to Plato's *Republic*, seeing has been a dominant metaphor for learning. In the latter text, Plato describes education as training to “look” in the right direction, thus equating curiosity with the figurative desire to see.

What stimulates such curiosity in a learner? Psychological accounts of curiosity posit that curiosity is piqued when we observe discrepancies (Berlyne, 1960; Oudeyer & Kaplan, 2009), perceive a moderate gap in our knowledge (Loewenstein, 1994), or expect the resolution of our curiosity to improve the utility of our beliefs (Dubey & Griffiths, 2017, 2020). Studies based on these theories have explored methods to stimulate curiosity (Baranes, Oudeyer, & Gottlieb, 2014; Dubey, Griffiths, & Lombrozo, 2019; Gentry et al., 2014; Law et al., 2016; Menon & Soman, 2002), and curiosity has in turn been linked with better learning (Von Stumm, Hell, & Chamorro‐Premuzic, 2011), memory (Gruber & Ranganath, 2019; Kang et al., 2009; Marvin & Shohamy, 2016), well‐being (Sakaki, Yagi, & Murayama, 2018), and decision‐making (Pierce, Distefan, Kaplan, & Gilpin, 2005). There thus lies tremendous value in identifying effective ways to promote curiosity.

In the current paper, we test a novel approach to stimulating curiosity: changing the social environment. A wealth of prior work suggests that our actions and decisions are influenced by social factors (Cialdini & Trost, 1998): Observing the behavior of other individuals or groups can affect an individual's thoughts, feelings, attitudes, and behaviors (Berns, Capra, Moore, & Noussair, 2010; Legare, Sobel, & Callanan, 2017). Research in marketing and social psychology shows that people rely on the judgments of others to infer the value of an action (Amblee & Bui, 2011; Moyer, Carson, Dye, Carson, & Goldbaum, 2015; Rao, Greve, & Davis, 2001). Research in education suggests that children's learning is informed not only by the material available to them, but also by the active work of other children and their social and cultural environment (Kashdan & Fincham, 2004; Parr & Townsend, 2002). As the Internet and social media become ever‐more pervasive, we can expect widespread effects of social cues on a variety of judgments and behaviors (Gruzd & Wellman, 2014; Kim & Srivastava, 2007).

On the basis of these findings, we explore the potential influence of a particular social cue—popularity—on people's curiosity regarding everyday questions about science. If popularity indeed affects curiosity, then interventions on the social environment could have important implications for curiosity researchers and for the design of effective learning environments.

## 2 Background

### 2.1 Theories of curiosity

Although curiosity has long been recognized as an important aspect of cognition, there is no single, agreed‐upon definition or account (Kidd & Hayden, 2015). Researchers differ in whether they differentiate curiosity from interest (Donnellan, Aslan, Fastrich, & Murayama, 2020; Grossnickle, 2016; Kashdan & Silvia, 2009) or subdivide curiosity into types (Berlyne, 1954). For our purposes we adopt a broad definition of curiosity as a drive state for information (Dubey & Griffiths, 2020; Kidd & Hayden, 2015; Liquin & Lombrozo, 2020; Murayama, FitzGibbon, & Sakaki, 2018), identified through a learner's self‐reported curiosity and subsequent behavior. Extant accounts make different predictions about the conditions under which this drive state is likely to be elicited.

Berlyne proposed one of the earliest theories of curiosity, according to which curiosity is triggered by incongruity and violation of expectations (Berlyne, 1960). Building on these ideas, Loewenstein proposed that curiosity is a state of deprivation prompted by a perceived gap in knowledge or understanding; the result is a desire to close the “information gap” between one's existing information set and a desired state (Golman & Loewenstein, 2015; Loewenstein, 1994), with a modest information gap prompting the greatest curiosity. More recently, Dubey and Griffiths (2017, 2020) proposed a rational model of curiosity which posits that curiosity is evoked whenever people perceive an opportunity to increase the value of their knowledge, that is, topics that either increase understanding or perceived usefulness. While theories continue to be tested and developed, these three accounts provide useful starting points for developing interventions on curiosity, and for considering why social cues might play a role.

### 2.2 Stimulating curiosity

Despite theoretical disagreements, curiosity is universally positively regarded (Gruber, Valji, & Ranganath, 2019; Jirout, Vitiello, & Zumbrunn, 2018; Von Stumm et al., 2011) and a number of studies have explored methods to stimulate curiosity. For instance, Berlyne's incongruity theory prompted a number of studies that stimulated curiosity by designing “optimally incongruent” stimuli (Berlyne, 1963; Nakatsu, Rauterberg, & Vorderer, 2005). Loewenstein's “information‐gap” theory has been used by many researchers to induce curiosity in education (Gentry et al., 2014; Pluck & Johnson, 2011), design (Law et al., 2016), and marketing (Menon & Soman, 2002). As one example, Law et al. (2016) showed that incomplete information (i.e., inducing an information gap) can be used to prompt curiosity and motivate participants in crowdsourcing tasks. Finally, the rational model of curiosity (Dubey & Griffiths, 2017, 2020), while relatively new, is consistent with work showing that a scientific topic's perceived usefulness is a strong predictor of an individual's curiosity and motivation to learn about it (Dubey et al., 2019; Liquin & Lombrozo, 2020; Rossing & Long, 1981).

### 2.3 The present research

Despite the sizable literature on curiosity, little work has explored methods to influence curiosity by manipulating the social environment. Important exceptions include studies of the social nature of curiosity focusing on the role of group membership (Sinha, Bai, & Cassell, 2017a; Thomas & Vinuales, 2017), and models of how curiosity is influenced in social and group learning settings (Paranjape, Bai, & Cassell, 2018; Sinha, Bai, & Cassell, 2017b). For example, Sinha et al. (2017a) used a data‐driven approach to identify behaviors that maximize an individual's probability of demonstrating curiosity during open‐ended problem‐solving in group work. While these studies shed light on the social nature of curiosity, they have not manipulated curiosity by leveraging the social environment. However, previous theories of curiosity suggest this can be achieved. For instance, other people's interest in a question could itself be a source of incongruity or surprise, thereby stimulating curiosity (Berlyne, 1963; Loewenstein, 1994). Given that other people's choices can affect perceptions of value (Berns et al., 2010), we might also expect that social cues, such as the popularity of a question, could indicate the value of knowing the answer, which in turn could influence curiosity (Dubey & Griffiths, 2017, 2020).

Guided by these ideas, this paper asks the following questions:

1. Do social cues influence curiosity? More specifically, are people more curious about the answer to a question that is high in popularity compared to one that is low in popularity?
2. Are people more likely to seek the answers to popular (vs. unpopular) questions, and is this in part because they are more curious about them?

Finally, we also consider a possible moderator of these effects—a learner's relative access to information—on the assumption that social cues are more likely to have a powerful effect (a) when alternative cues are poor, and (b) when social informants have access to more information than the learner.

Answering these questions provides an opportunity to empirically evaluate theoretical claims related to the link between the social environment and curiosity while also extending the rich literature in psychology on social influence.

## 3 Experiment 1

In Experiment 1, we investigated whether popularity affects people's curiosity in a situation with *impoverished information* (i.e., one with few additional cues, and limited information relative to informants), and whether a shift in curiosity affects the information people subsequently choose to reveal. The experiment tested the following predictions: (a) Participants will report greater curiosity about popular questions relative to unpopular questions; and (b) Participants will be more likely to reveal the full questions and answers for popular questions relative to unpopular questions.

### 3.1 Method

#### 3.1.1 Participants

Three hundred participants were recruited from Amazon Mechanical Turk (AMT) and were paid $1.00 for their participation in a 7–8 min study. Participation was restricted to AMT workers with an IP address within the United States. For this experiment and all that follow, informed consent was obtained using a consent form approved by the Institutional Review Board (IRB) at the University of California, Berkeley. While demographic information was not collected, we expect the characteristics of our sample to track the general AMT population (e.g., Paolacci & Chandler, 2014).

#### 3.1.2 Stimuli

The stimuli used in the experiment were 50 why‐ or how‐ questions sampled from Reddit's *Explain Like I'm Five* subreddit, collected over the course of 4 months. We chose questions that were moderately popular, as reflected in up‐votes between 200 and 600, to avoid outliers in either direction. For each question, we manually identified the main topic. For example, for the question “How does the body separate water from stomach acid?,” we identified the topic as “Digestion.” (See Appendix S1 for all items.)

#### 3.1.3 Procedure

**Fig. 1:**
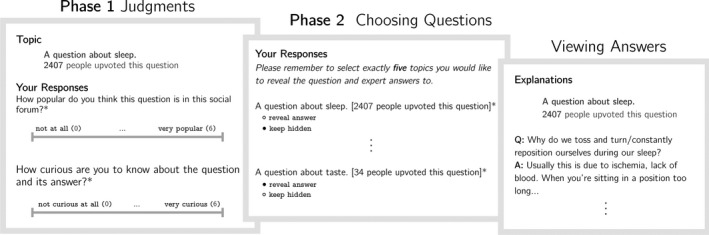
Design of Experiment 1. The experiment was divided into two phases. In Phase 1, participants were presented with the topics and number of up‐votes for each of 10 questions and asked to rate their curiosity and perception of the question's popularity. In Phase 2, participants had the choice to reveal the questions and answers for five of the previously shown question‐topics. Finally, the selected questions and answers were revealed. Note that instructions were provided before each phase.

At the start of the experiment, each participant was assigned to 10 questions randomly sampled from our 50‐question database. The experiment was then divided into two phases, described below (see also Fig. 1).

##### 3.1.3.1 Phase 1

In the first phase, participants were presented with each question, but indicated only in terms of its topic (e.g., “a question about digestion”). They were also presented with the number of up‐votes that the question putatively received on a “popular online forum.” They were told to note that “the up‐votes were given by members of the online community who viewed the full text, not just the topic of the questions,” and that “the up‐votes were only based on the questions and not the answers to those questions.” Thus participants had access to *less* information than those who provided the votes.

Of the 10 questions presented to each participant, five were randomly assigned a high number of up‐votes, and five a low number of up‐votes. These numbers were drawn from low‐variance normal distributions with means of 2,405 and 24, respectively. After seeing each question and its corresponding number of up‐votes, participants were asked to rate, on a scale from 0 to 6, “How popular do you think this question is in this social forum?” This question was a manipulation check to ensure that participants correctly interpreted the number of up‐votes. Participants also rated their curiosity (“How curious are you to know about the question and its answer?”), again on a scale from 0 to 6. This was the key variable of interest in Phase 1.

##### 3.1.3.2 Phase 2

In the second phase, participants could reveal the questions and answers corresponding to five of the 10 question‐topics rated in Phase 1. The question‐topics and up‐votes from Phase 1 were again presented, and participants indicated their five choices. The corresponding questions and answers were then revealed.

### 3.2 Results

#### 3.2.1 Phase 1

**Fig. 2:**
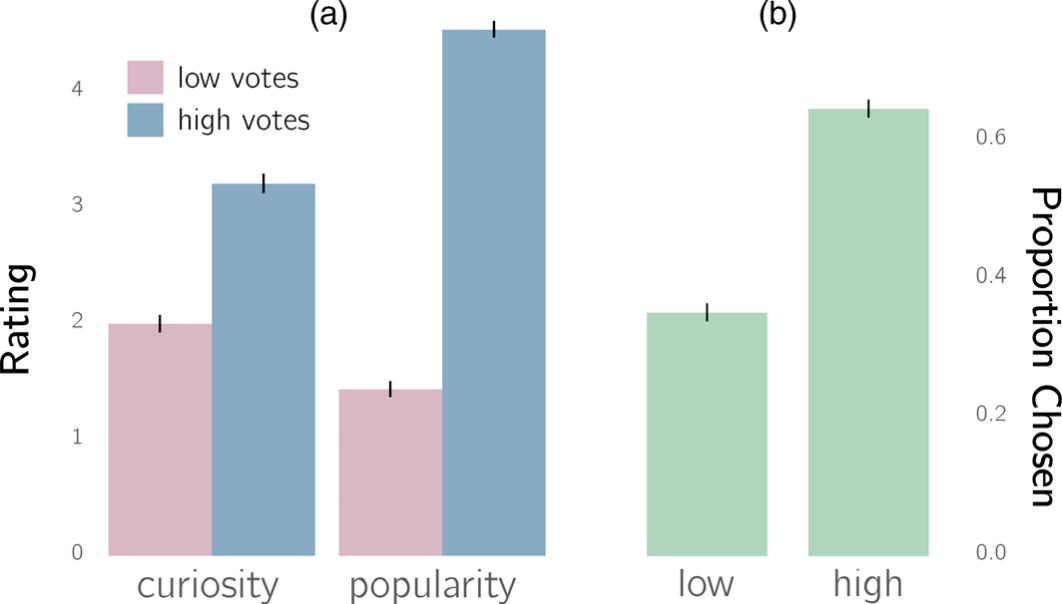
Popularity influences people's curiosity (Experiment 1). (a) Participants rated question‐topics that were presented with higher numbers of up‐votes as more popular, and reported higher levels of curiosity about the questions and their answers. (b) Participants were more likely to reveal the full questions and answers for questions with higher numbers of up‐votes. Error bars represent the standard error of the mean for the different ratings.

We first confirmed that our manipulation of up‐votes successfully manipulated perceived popularity. As shown in Fig. 2a, the mean popularity of questions with high up‐votes was significantly higher (by 3.18 points) than that of low up‐vote questions, *t*(299) = −30.4, *p* < .001 (paired‐samples *t*‐test). We next tested whether our popularity manipulation influenced participants' curiosity. As shown in Fig. 2a, mean curiosity for questions with high up‐votes was significantly higher (by 1.23 points) than that for questions with low up‐votes, *t*(299) = −14.1, *p* < .001 (paired samples *t*‐test).

#### 3.2.2 Phase 2

To evaluate whether participants were more likely to reveal the full questions and answers for high up‐vote questions, we tested whether high up‐vote questions were revealed more often than the chance value of 50%. As shown in Fig. 2b, participants revealed high up‐vote questions 64.3% of the time, which was greater than chance, *t*(299) = 11.0, *p* < .001 (single‐sample *t* test).

Finally, we considered whether curiosity mediated the effect of up‐votes on whether a question–answer pair was revealed. We first ran a logistic regression predicting whether a question was chosen from the experimental manipulation of up‐votes. Encoding low and high as 0 and 1, respectively, this yielded a significant and positive coefficient of 1.18 (*z* = 15.48, *p* < .001). We next considered a regression predicting whether a question was chosen from rated curiosity, yielding a significant and positive coefficient of 0.50 (*z* = 21.82, *p* < .001). We then fit a multiple regression with both curiosity and up‐vote condition as predictors: This yielded coefficients of 0.45 and 0.80, respectively (*z* = 19.10, *p* < .001 and *z* = 9.63, *p* < .001), suggesting partial mediation, as confirmed by a Sobel test (*z* = 12.36, *p* < .001). These findings are consistent with the idea that up‐votes affected whether a question/answer was revealed in part because they affected curiosity about that question/answer.

### 3.3 Discussion

Experiment 1 tested and found support for two predictions about the effects of popularity in an impoverished information environment. Participants were more curious about popular questions (vs. unpopular questions), and they were more likely to reveal the full questions and answers for popular (vs. unpopular) questions. Moreover, the effect of popularity on revealed information was partially mediated by curiosity.

## 4 Experiment 2

Experiment 2 had three aims. First, we investigated whether our predictions for Experiment 1 generalize to a situation with *rich information*. Participants received the full text for each question (vs. its topic), so their information matched that of the putative members of the online forum who provided up‐votes.

Second, we investigated *why* popularity might affect curiosity. Based on extant theories of curiosity, we considered four hypotheses and incorporated additional measures to test the corresponding factors. First, up‐votes could change participants' beliefs about their own knowledge, leading to an information‐gap (Dubey & Griffiths, 2017; Loewenstein, 1994). For example, seeing that a question has many up‐votes could make participants question their initial confidence in knowing the answer. To test this, we asked participants to rate their *confidence* in knowing each answer. Second, up‐vote information could prompt curiosity by introducing an incongruity between participants' expectations and actual up‐votes, again introducing an information‐gap (in this case, concerning why others did or did not up‐vote). For this, we asked participants to rate how *surprised* they were by each question's popularity. Third, participants might infer that knowing the answers to high up‐vote questions would be valuable socially. Correspondingly, we asked participants to rate the *social utility* of knowing each answer. Fourth, participants might infer that knowing the answers to the questions with high up‐votes would be of more general value. To test this, we asked participants to rate how *useful* they thought knowing each answer would be in the future.

The third aim of the experiment was to introduce control conditions. First, we included a “post‐number” control in which participants received high and low post‐numbers (vs. high and low upvotes). This was included to test whether up‐votes were driving effects because of the social content they conveyed, not merely because they introduced high versus low numbers. Second, we included a “baseline” control in which questions were not accompanied by a number. The goal was to evaluate whether high up‐votes *raised* curiosity, low up‐votes *lowered* curiosity, or both.

### 4.1 Method

#### 4.1.1 Participants

Six hundred participants were recruited from AMT and paid $1.50 for participation in a 12‐min study. We removed participants who failed a simple attention check (a short quiz at the end to assess whether participants understood the instructions). Eight participants were thus excluded, but their inclusion does not affect the significance of our findings. The final sample consisted of 592 participants, who were assigned to the *up‐vote* condition with .50 probability (297 participants) or one of the two control conditions, each with .25 probability: *post‐number* (161 participants) or *baseline* (134 participants).

#### 4.1.2 Stimuli

Stimuli were the same 50 questions in Experiment 1.

#### 4.1.3 Procedure

The design and procedure followed Experiment 1, with two key differences. First, participants received each question in full (not only the topic). Second, participants provided the following additional ratings on 0–6 scales: confidence (“How confident are you that you know the correct answer to this question?”), surprise (“How surprised are you by the popularity of this question?”), social utility (“To what extent would knowing the answer to this question be useful to you in a social setting?”), and usefulness (“To what extent would knowing the answer to this question be useful to you in the future?”).

In the *up‐vote* condition, participants were presented with low or high up‐votes. In the *post‐number* condition, participants were presented with low or high post‐numbers, with numbers matching the distributions for up‐votes. Finally, in the *baseline* condition, participants received only the question text (with no numerical information). Note that for both the *post‐number* and *baseline* conditions, participants were not asked to rate their surprise, as they were not presented with up‐vote information.

### 4.2 Results

#### 4.2.1 Up‐vote condition

##### 4.2.1.1 Phase 1

**Fig. 3:**
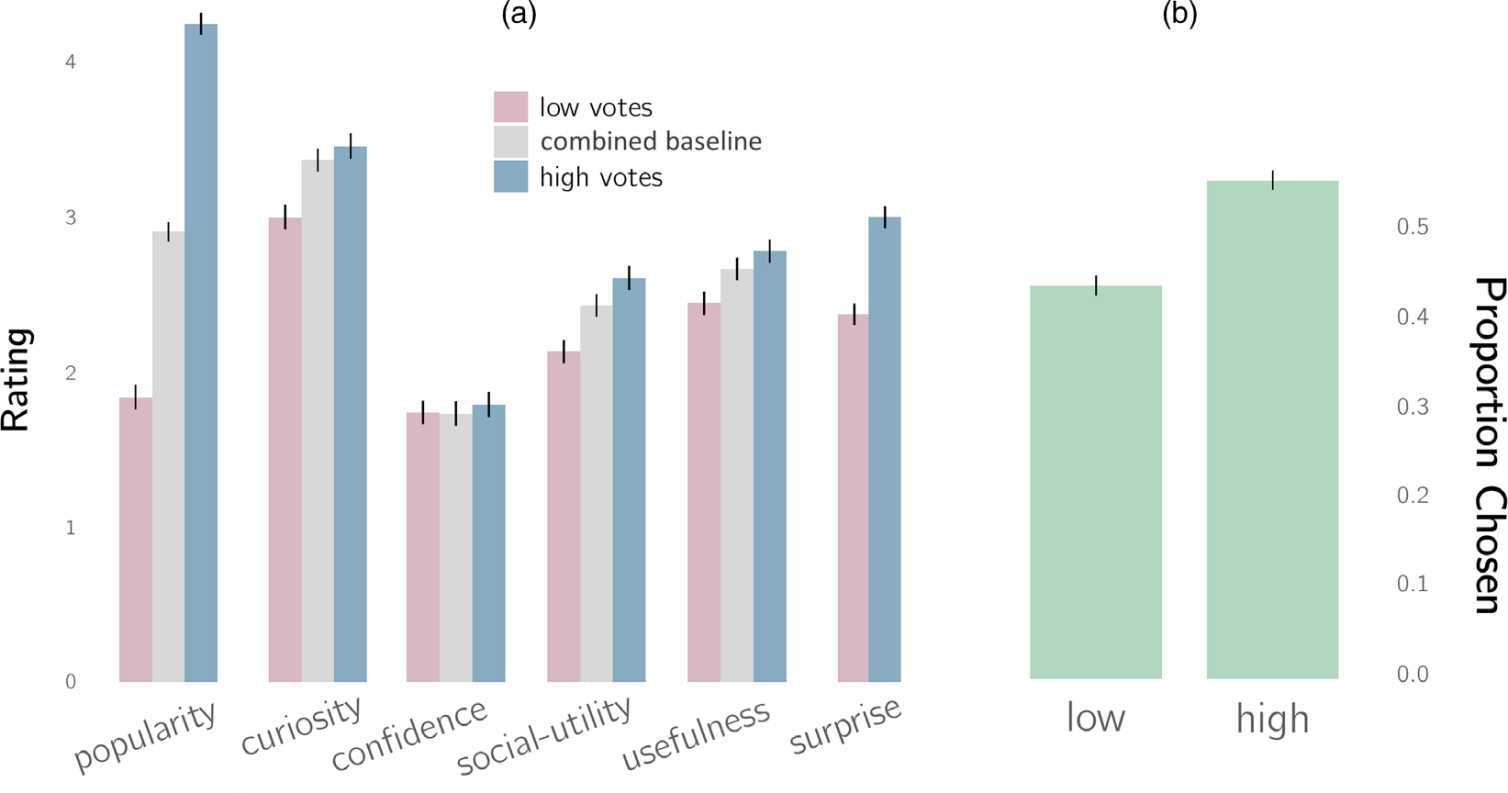
Popularity influences people's curiosity in a rich information environment (Experiment 2). (a) Up‐votes influenced participants' ratings for every judgment except for their confidence (see Table 1). (b) Questions with more up‐votes were once again more likely to be revealed by participants in Phase 2. Error bars represent the standard error of the mean for the different ratings.

We first tested whether up‐votes affected perceived popularity. As shown in Fig. 3a, high up‐vote questions were rated significantly more popular (by 2.39 points) than low up‐vote questions, *t*(296) = −21.5, *p* < .001 (paired‐samples *t*‐test).

Next, we tested whether curiosity was affected by up‐votes. As shown in Fig. 3a, curiosity was significantly higher (by 0.46 points) for questions with high up‐votes versus low up‐votes, *t*(296) = −7.14, *p* < .001 (paired‐samples *t*‐test). Paired‐samples *t*‐tests for each remaining judgment revealed that popularity did not reliably affect confidence, but it did have a significant effect on surprise, social utility, and usefulness, all of which were reliably correlated with curiosity (see Table 1).

**Table 1:** Impact of manipulating upvotes on judgment ratings (Experiment 2). “Curiosity correlation” refers to the correlation between each rating type and curiosity. Mean differences for each rating type were computed by subtracting the low up‐vote means from the high up‐vote means; correlations/differences significant at the .05 level are starred

Judgment	Curiosity Correlation	Mean Difference	*t*(296)	*p*‐Value
Popularity	0.35*	2.39*	−21.5	<.001
Confidence	0.01	0.05	−0.89	.37
Surprise	0.13*	0.63*	−7.27	<.001
Social utility	0.56*	0.47*	−8.71	<.001
Usefulness	0.61*	0.34*	−5.59	<.001

To evaluate whether the effect of up‐votes on curiosity was driven by these additional factors, we conducted a series of mediation analyses, which revealed that surprise, social utility, and usefulness were only partial mediators (see Appendix S1 for analyses). We also considered whether up‐vote condition explained variance above and beyond these additional factors. To do so, we conducted a multiple regression using up‐votes, confidence, surprise, social utility, and usefulness to predict curiosity. Up‐vote condition remained significant (0.13, *z* = 2.2, *p* < .05). Also noteworthy is that usefulness outperformed all other predictors with a coefficient of 0.50 (*z* = 22.62, *p* < .05 vs. −.18 for confidence, *z* = −11.9, *p* < .05 vs. .06 for surprise, *z* = 4.16, *p* < .05 vs. .28 for social utility, *z* = 12.20, *p* < .05).

##### 4.2.1.2 Phase 2

Participants revealed the answers to high up‐vote questions 54.75% of the time, significantly more often than chance, *t*(296) = 4.25, *p* < .001 (one‐sample *t*‐test, refer to Fig. 3b). Even in a rich information environment, manipulating up‐votes affected the information participants preferred to receive.

As in Experiment 1, we tested whether the effect of up‐votes on information revealed was mediated by curiosity. First, a logistic regression predicting question choice from the manipulation of up‐votes revealed a significant coefficient of 0.45 (*z* = 6.12, *p* < .001). A similar regression with curiosity as the predictor produced a coefficient of 0.38 (*z* = 18.79, *p* < .001). Next, in a multiple regression with both curiosity and up‐vote condition, the up‐vote coefficient was reduced to 0.33 (*z* = 4.21, *p* < .001), while curiosity remained comparable at 0.38 (*z* = 18.39, *p* < .001). This suggests that the effect of up‐votes on information revealed was partially mediated by curiosity, as confirmed by a Sobel test (*z* = 5.90, *p* < .001). Finally, we considered whether the effect of curiosity was still significant, controlling for all other judgments. We conducted a multiple regression using all six judgments to predict whether a question was revealed and found that curiosity outperformed all other predictors with a coefficient of 0.32, maintaining its significance (*z* = 11.76, *p* < .001, see Appendix S1 for full analysis).

#### 4.2.2 Post‐number condition

##### 4.2.2.1 Phases 1 and 2

In Phase 1, participants' curiosity did not vary as a function of post‐number condition, *t*(160) = 0.37, *p* = .71, nor did post‐numbers affect other judgments (*p* > .35). In Phase 2, questions with high post‐numbers were revealed 49% of the time, which was no higher than chance, *t*(160) = −1.14, *p* = .26 (single‐sample *t*‐test). These effects differed significantly from those in the up‐vote condition (with the exception of comparable null effects for confidence; see Appendix S1 for analyses) and suggest that the effects of up‐votes were not driven merely by their numerical content.

#### 4.2.3 Baseline condition

The aim of this condition was to explore how curiosity in the up‐vote condition differed from participants' baseline curiosity. First, we note that none of the ratings from the post‐number and baseline conditions were significantly different from each other and therefore we combined ratings from these two conditions. For participants in the up‐vote condition, we created two mean curiosity ratings, one in response to questions presented with low up‐votes, and one in response to questions presented with high up‐votes. We then compared these means to the combined ratings for participants in the post‐number and baseline conditions, and found that the mean curiosity rating in response to low up‐vote questions was significantly lower (by 0.37 points) than that for participants in the combined baseline condition, *t*(885) = −3.79, *p* < .001, while the mean rating in response to high up‐vote questions was not significantly different, *t*(885) = −0.91, *p* = .36 (Fig. 3). We found a similar effect for all other ratings (refer to Appendix S1).

In an additional experiment (*N* = 562, see Appendix S1, Study 2b), we considered the possibility that ratings in the high up‐vote condition did not differ from baseline because the up‐vote numbers were insufficiently high. We therefore replicated Phase 1 from the up‐vote and baseline conditions of Experiment 2, but with high up‐vote values drawn from a distribution with a higher mean (24,050 upvotes vs. 2,405). This succeeded in creating a more dramatic effect on popularity for questions with high up‐votes, yet we replicated the findings of Experiment 2: Curiosity ratings in response to the low up‐vote questions differed significantly from baseline; curiosity ratings for high up‐vote questions did not.

### 4.3 Discussion

The findings from Experiment 2 replicate our findings from Experiment 1 in an environment with rich information. Even though participants had access to the full content of each question, popular questions induced greater curiosity than unpopular questions, participants were more likely to reveal their answers, and the effect of popularity on information revealed was partially mediated by curiosity (see also Experiment 2c in Appendix S1 for an additional replication). Furthermore, these results were driven specifically by up‐vote information (post‐number had no significant effects), and predominantly by a reduction in curiosity toward low up‐vote questions. Finally, we also found that the effects of curiosity were not fully captured by ratings of confidence, surprise, social value, or utility, suggesting that extant accounts of curiosity cannot readily explain our results.

## 5 Comparison of Experiments 1 and 2

We compared effects across Experiments 1 (impoverished information) and 2 (rich information) to better understand whether relative access to information moderates the effects of social cues on curiosity. In Experiment 1, manipulating up‐votes increased curiosity by +1.23 points, which was significantly greater than the increase of +0.46 points in Experiment 2, *t*(595) = 7.24, *p* < .001. Similarly, although participants chose questions with high up‐votes more frequently in both experiments, this proportion was significantly higher in Experiment 1 (64.7%) than in Experiment 2 (54.8%), *t*(595) = 5.48, *p* < .001. These results suggest a greater role for social cues in driving curiosity and revealed information when alternative cues are weak and/or the learner's information is impoverished relative to social informants.

## 6 General discussion

We began this paper by asking whether a learner's social environment can influence curiosity. The answer is “yes”: Manipulating the perceived popularity of a question influenced participants' curiosity about the answer to that question, and the information participants chose to reveal. These findings contribute to a growing body of research demonstrating the power of social cues on subsequent judgments and decisions.

Our results suggest that the primary role of popularity was to reduce curiosity about less popular questions, rather than to increase curiosity about more popular questions. Perhaps popularity serves as a cue for deciding what *not* to obtain further information about, as opposed to a cue for deciding what to pursue. That is, unpopularity could successfully eliminate options, whereas popularity may not be sufficient to elevate options already under consideration. That said, popularity could potentially have more dramatic effects in an environment in which the same participant receives positive popularity cues for some items but not for others (for instance, in learning about movies for which only some have available reviews), in contrast to our between‐subjects design. Additionally, effects could be more dramatic and bidirectional in domains other than science, for which social relevance might have greater consequences.

Our findings also have implications for theories of curiosity. First, we find that up‐votes affected curiosity by inducing surprise about why other people up‐voted the question (which was a feature of the social environment). This result is interesting because it extends the information‐gap hypothesis to a previously untested type of cue, and it confirms that an information gap can be produced not just by the content of a question, but by contextual information. Second, our results highlight the importance of perceived usefulness and social utility in influencing curiosity, thereby supporting more recent accounts of curiosity that highlight the value of information (Dubey & Griffiths, 2017, 2020). Finally, it is worth highlighting that the manipulation of social context is extrinsic to the learner. While there is a line of research that characterizes curiosity purely as an intrinsic drive, our work identifies extrinsic influences on this drive, raising new questions about how extrinsic and intrinsic cues interact. This is also in line with more recent proposals for curiosity that do not aim to explicitly distinguish between intrinsic and extrinsic drivers of curiosity, instead focusing on the characteristics and consequences of the drive for information itself (Dubey & Griffiths, 2020; Kidd & Hayden, 2015; Murayama et al., 2018).

The COVID‐19 pandemic has heightened existing concerns about people's ability to direct their own learning toward accurate information, and it has done so in an environment that makes virtual social cues—such as up‐votes—all the more pervasive. Our findings are therefore especially timely, and they point to both the promise and dangers of online social cues in a complex environment in which people must navigate competing social cues and misinformation. The role of curiosity in this process is an important direction for future research.

Despite the promise of our results, the impact of our study is limited by the nature of our stimuli and task: We focused on short‐term consequences of manipulating popularity about science questions through up‐votes. While up‐votes have natural analogs on social media and in online learning environments, it is less clear how they might translate into a more traditional classroom or to other kinds of learners (such as children). Investigating a broader range of materials and cues, as well as variation across learners, are important directions for future research, as is measuring learning outcomes that could have real‐world consequences for formal or informal learning. Having documented a new phenomenon, we need to better understand its boundary conditions and potential applications.

In Plato's allegory, the purpose of education is to redirect an individual's “sight.” Our findings suggest that manipulating the social environment is one way that educators can help learners figure out where to look.

## Authors' contributions

Conceptualization: R.D., H.M., and T.L. Methodology: R.D., H.M., and T.L. Software: R.D and H.M. Investigation: R.D., H.M., and T.L. Writing: R.D., H.M., and T.L. All authors approved the final version of the manuscript for submission.

### Open Research badges

This article has earned Open Data and Open Materials badges. Data and materials are available at https://github.com/rach0012/Social_curiosity.

## Supporting information


**Appendix S1:** Experimental materials.
