# Supplementary material for: Curiosity Is Contagious: A Social Influence Intervention to Induce Curiosity
Source: Cogn Sci. 2021 Feb 12;45(2):e12937. doi: 10.1111/cogs.12937 (PMC7900967; doi:10.1111/cogs.12937)
Supplement: Supplementary file 1 — Appendix S1: Experimental materials. [file COGS-45-e12937-s001.pdf]

Curiosity is Contagious: A Social Influence Intervention to Induce Curiosity

Supplemental Material

Rachit Dubey[1]

Department of Computer Science,

Princeton University

Hermish Mehta[1]

Department of Electrical Engineering and Computer Sciences,

University of California, Berkeley

Tania Lombrozo

Department of Psychology,

Princeton University

[1]These authors contributed equally

Corresponding author: Rachit Dubey. Email: *rdubey@princeton.edu*

Curiosity is Contagious: A Social Influence Intervention to Induce Curiosity

Supplemental Material

Experimental Materials

Instruction text for Experiment 1

Phase 1. "On the following pages, we will show you 10 questions people have asked and number of upvotes those questions got on a popular online forum. However, we will only show you the topic of the question, and not the full text. **Note that the upvotes were given by members of the online community who viewed the full text, not just the topic of the questions. The upvotes were only based on the questions and not the answers to those questions.** For each question, we will ask you to make a series of judgments. Please press the next button below or the arrow key to proceed."

Phase 2. "We will now give you the chance to know the answers to the previous questions. If you could see the questions (and answers experts wrote), for five of these questions, which five would they be? In the next page, you will be asked to select 5 topics of the 10 that you have been presented with. Please select exactly five topics, otherwise the page will not let you submit. Please press the next button below or the arrow key to proceed."

Instruction text for Experiment 2

Phase 1 (upvote condition). "On the following pages, we will show you 10 questions people have asked and number of upvotes those questions got on a popular online forum. **Note that the upvotes were given by members of the online community who viewed just the question, not the answer. The upvotes were only based on the questions and not the answers to those questions.** For each question, we will ask you to make a series of judgments. Please press the next button below or the arrow key to proceed."

Phase 1 (control conditions). "On the following pages, we will show you 10 questions people have asked on a popular online forum. For each question, we will ask you to make a series

of judgments. Please press the next button below or the arrow key to proceed."

Phase 2 (all conditions). "We will now give you the chance to read the answers to the previous questions. If you could see the explanations experts wrote for five of these questions, which five would they be? In the next page, you will be asked to select 5 questions of the 10 that you have been presented with. Please select exactly five questions, otherwise the page will not let you submit. Please press the next button below or the arrow key to proceed."

Judgment ratings

Experiment 1. In Experiment 1, participants responded to each of the following on a scale of 0–6:

1. *Curiosity*: "How curious are you to know about the question and its answer?"
2. *Popularity*: "How popular do you think this question is in this social forum?"

Experiment 2. In Experiments 2 and 2b, participants responded to each of the following on a scale of 0–6:

1. *Curiosity*: "How curious are you to know the answer to this question?"
2. *Popularity*: "How popular do you think this question is in this social forum?"
3. *Confidence*: "How confident are you that you know the correct answer to this question?"
4. *Surprise*: "How surprised are you by the popularity of this question?"
5. *Social utility*: "To what extent would knowing the answer to this question be useful to you in a social setting?"
6. *Usefulness*: "To what extent would knowing the answer to this question be useful to you in the future?"

Stimuli

Table S1 contains all the fifty questions that were sampled from Reddit's *Explain Like I'm Five* subreddit. In Experiment 1, participants were shown the topics of the questions (right) and in Experiment 2 and 2b, they were shown the full text of the questions (left).

Table S1

All the 50 questions collected from Explain Like I'm Five (left) and topics of those questions (right).

Questions	Topics
Why do your eyelids get puffy after crying?	Eyelids
Why aren't other animals as freaked out by bugs and creepy crawlies as humans?	Insects
Why do some alcoholics suffer life threatening withdrawal symptoms, while others with the same drinking habits don't when they quit?	Alcoholism
How do scientists know what the global temperature was millions of years ago?	Climate study
Why is the consistency of my ice cream different when it melts and I refreeze it?	Ice-cream
How does the body separate water from stomach acid?	Digestion
Why is CPR for drowning different than CPR for people who collapse from heart problems? (e.g rescue breaths are recommended for one but not the other)	CPR
What make some objects 'bouncier' than others?	Object physics
What gives something it's taste? Does pyrite taste like table salt because they are both cubes?	Taste
Why do typical spray pump bottles of cologne or perfume evaporate quickly when left on their side or "tipped over," but not while upright?	Perfume
Why do car windows get a grid pattern on them	Cars
Why is math (statistics, calculus, etc) so important for a strong programmer?	Math
Continued on next page	

Table S1 – continued from previous page

Questions	Topics
What makes something microwaveable or non-microwaveable? Is all food microwaveable?	Microwaves
How do they get the liquid medicine to completely fill liquicaps, like Dayquil?	Medicine
How do such small doses of things like cocaine and heroin kill you? How do these small powders have such a big effect on your body?	Drugs
What causes those pulled neck muscles that happen from doing nothing like yawning or rolling in your sleep?	Muscles
What is the difference between forward and reverse osmosis?	Osmosis
How does sleep restore the body's energy?	Energy
How do our lungs prevent or eliminate dust?	Lungs
What exactly is happening when we blur our eyes on command?	Blur
Why are bodies able to create an entire body with it's own lifetime supply of regenerative cells, but is itself unable to prevent gradual decay over a lifetime?	Cells
How does rabies induce hydrophobia?	Rabies
How can certain animals such as frogs and flies freeze solid and survive, but most mammals suffer extreme tissue damage?	Mammals
How do breeders ensure diversity among their animals' offspring? Wouldn't they have to constantly buy new breeding pairs?	Animal breeding
If rockets use controlled explosions to propel forward, why can't we use a nuclear reaction to launch/fly our rockets?	Rockets
How can alcohol withdrawal or detox kill you?	Alcohol
Why do tongues get weird bumps when burnt or after eating something really sweet or really salty?	Tongue
Continued on next page	

Table S1 – continued from previous page

Questions	Topics
Do multivitamins and Omega-3 pills actually do anything? Or is it more of a placebo-type thing?	Multivitamin
How do flares stop missiles?	Missiles
Why is it that drinking fizzy drinks, even if they don't touch your teeth, is harmful to your teeth?	Teeth
Why does giving someone a transfusion of my blood to someone not give them my immunity?	Blood transfusion
Why can't the asteroid belt accumulate into one rocky planet?	Asteroids
How do earphones produce adequate bass despite their size?	Earphones
An anechoic chamber at Orfield Laboratories in Minnesota has negative decibel levels (lower than -9db). How is this possible?	Sound
How do car dealerships make money when they claim the markup on new cars is only a few hundred dollars?	Car dealership
Why do US based airlines lag behind in service and quality, especially in their premium cabins?	Airlines
What are the biological advantages and disadvantages of trees shedding their leaves vs keeping them all year round (deciduous vs coniferous)?	Trees
What determines where a person will store the excess fat? Why it differs from person to person?	Bodyfat
Why are 9mm bullets less dangerous than 7.62 or even 5.56 ones? Shouldn't they deal more damage with bigger size?	Bullets
When bacteria die, for example when boiling water, where do their corpses go?	Bacteria
What happens that makes beer taste terrible after warming up and then re-chilling? What makes beer 'skunky'?	Beer
Continued on next page	

Table S1 – continued from previous page

**Questions**	**Topics**
What is the difference between beat, bar, steps, tempo, tact, and rhythm?	Music
Why does shampoo not lather up well when you shampoo for the first time in a while?	Shampoo
Why is therapy effective? What is it about the brain that allows talking about your problems to help fix them?	Therapy
Why do we toss and turn/constantly reposition ourselves during our sleep? What makes one position suddenly stop being comfortable even when we are not fully conscious?	Sleep
What is the difference between time signatures that have the same ratio?	Tempo
What's special about CO2 that we add it to water/soda and not other gasses? Why not use compressed air or another gas?	Aerated drinks
What occurs physically that causes a person to foam at the mouth?	Foaming
Why is it hard to implement a standard volume across various mediums like radio and television?	Radio
Why waves? All energy transfer in nature from one point to another happens in waves. Light, sound, even gravity travels in waves. Which fundamental property of nature is responsible for wave-like nature? Are there other non-wave like ways to transfer energy from one point to another?	Waves

Means and standard errors for the graphs in the main paper

In the below tables, we report the mean and standard errors for the plots shown in Figure 2 and Figure 3 in the main paper.

judgment	low votes	high votes
curiosity	2.0±0.08	3.21±0.08
popularity	1.44±0.07	4.53±0.07

Table S2

Table containing the mean values and standard errors for Figure 2a in the main paper

	low votes	high votes
proportion chosen	0.36 ±0.01	0.64±0.01

Table S3

Table containing the mean values and standard errors for Figure 2b in the main paper

judgment	low votes	combined baseline	high votes
popularity	1.83±0.08	2.89±0.05	4.22±0.07
curiosity	2.98±0.08	3.35±0.06	3.44±0.08
confidence	1.73±0.08	1.72±0.05	1.78±0.08
social-utility	2.12±0.07	2.42±0.05	2.60±0.08
usefulness	2.43±0.08	2.65±0.06	2.77±0.08
surprise	2.36±0.07		2.99±0.07

Table S4

Table containing the mean values and standard errors for Figure 3a in the main paper

	low votes	high votes
proportion chosen	0.44 ±0.01	0.56±0.01

Table S5

Table containing the mean values and standard errors for Figure 3b in the main paper

Experiment 2b

The aims of this experiment were twofold. First, we aimed to test whether the effect of high upvotes would be stronger if the high upvotes were drawn from a distribution with a higher mean.

Second, we again aimed to obtain people's curiosity ratings in the absence of upvotes (similar to the baseline condition of Exp 2) and thereby assess whether upvotes lowered or raised curiosity relative to the baseline.

Participants. 562 participants were recruited from AMT and paid \$1.40 for participating in a 10-minute study. We removed participants who failed a simple attention check at the end of the experiment. 41 participants were excluded on this basis, but their inclusion does not affect the significance of our findings. The final sample consisted of 521 participants, who were randomly assigned to the *upvote* condition (243 participants) or the *baseline* condition (278 participants).

Stimuli. Stimuli were the same fifty questions used in Experiment 1 and 2.

Method

The design and procedure followed Experiment 2, with the following differences. First, high upvote numbers were drawn from a normal distribution with a much higher mean of $24,050$ (vs. 2405 in Exp 1 and 2). The low upvote numbers were drawn from the same distribution as in Exp 1 and 2 (mean=24). Second, this experiment included only Phase 1.

Results

Upvote Condition

As a manipulation check, we first confirmed that popularity was higher for high upvote questions relative to the low upvote questions ($t(242) = -20.3, p < .001$; see Figure S1). We next evaluated whether Experiment 2b succeeded in increasing high popularity relative to Experiment 2. An ANOVA with experiment (Experiment 2, Experiment 2b) and condition (high upvotes, low upvotes) as independent variables, and popularity as the dependent variable, revealed a significant interaction, $F(1) = 3.11, p < .001$: the popularity of high upvote questions in Experiment 2b was significantly higher than that for Experiment 2 ($t(538) = -2.35, p < 0.05$), whereas the popularity of low upvote questions did not differ across experiments ($t(538) = -0.23, p = 0.82$).

We next evaluated whether the effects found in Phase 1 of Experiment 2 were successfully replicated in Experiment 2b. They were – most notably, upvotes had a significant impact on

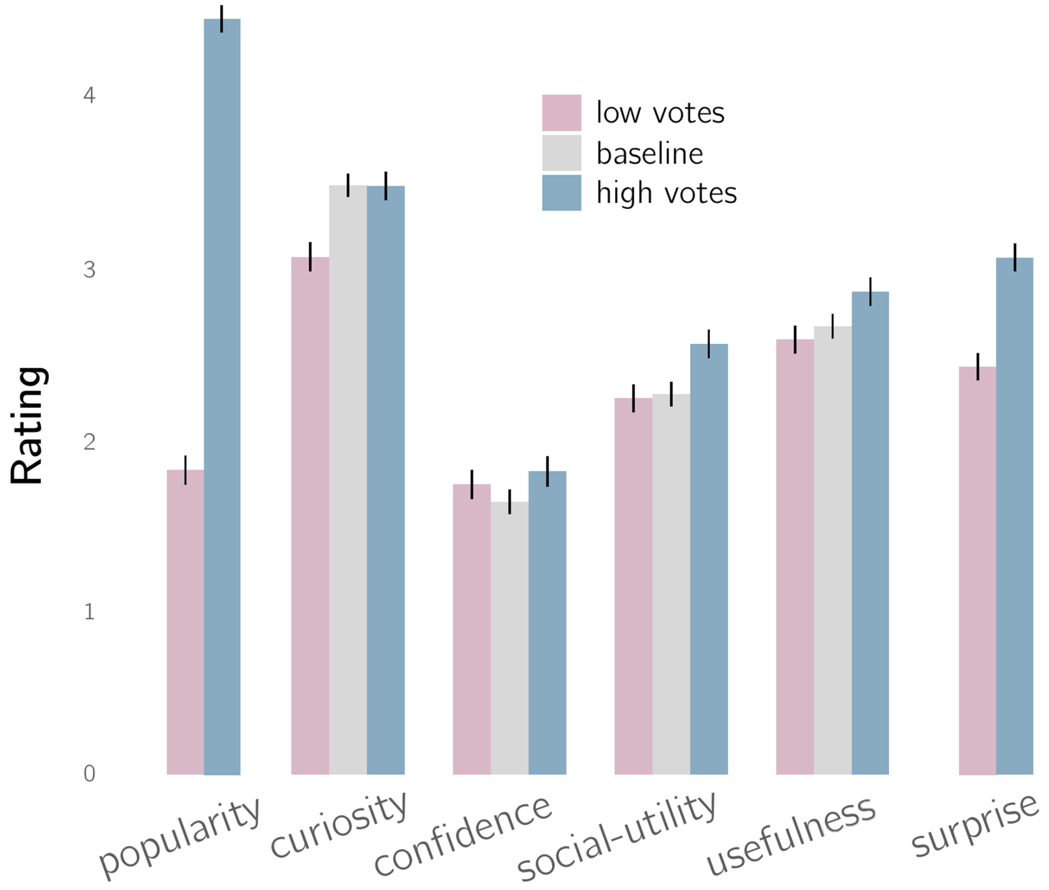

Figure S1. **The effect of popularity on curiosity is not stronger if the high upvotes are drawn from a distribution with a higher mean (Experiment 2b)**. There was no significant difference between the ratings (except popularity) between Exp 2 and 2b. Error bars represent the standard error of the mean for the different ratings.

curiosity, with a linear regression predicting curiosity from upvote condition revealing a significant co-efficient of 0.42 ($z = 3.47, p < .001$), and this effect remained significant when taking confidence, surprise, social utility, and usefulness into account, with usefulness accounting for the most variance (co-efficient = 0.73 ($z = 11.64, p < .001$)).

Comparison to Baseline Condition

Curiosity for low upvote questions was significantly lower than baseline curiosity, $t = -3.8, p < 0.001$ (two-sample t-test). However, curiosity for high upvote questions was not

significantly higher than baseline curiosity, $t = 0.03, p = 0.98$ (two-sample t-test). This suggests that low upvotes successfully reduced curiosity, but does not provide evidence that high upvotes increased curiosity (Figure S1).

Discussion

Findings from Exp 2b suggest that the effect of popularity on curiosity is not stronger if the high upvotes are drawn from a distribution with a higher mean. Furthermore, these findings support the findings from Exp 2 and suggest that the dominant effect of upvotes was that it reduced the curiosity for unpopular questions, rather than increase curiosity for popular questions.

Additional results for Experiment 2

Upvote Condition

Phase 1. While participants' judgements of surprise, social utility, and usefulness were all correlated with curiosity, they only partially mediated the effect of upvotes on curiosity. We now provide details of this analysis. First, a linear regression predicting curiosity from the manipulation of upvotes revealed a significant, positive coefficient of 0.45 ($z = 6.17, p < .001$). A similar regression with confidence as the predictor produced a coefficient of 0.01, but it was not significant ($z = 0.60, p = 0.5$), thereby suggesting that the effect of upvotes on curiosity was not mediated by confidence. We also performed similar regressions with surprise, social utility, and usefulness as predictors, which produced coefficients of 0.14 ($z = 7.17, p < 0.001$), 0.63 ($z = 36.73, p < 0.001$), and 0.66 ($z = 42.31, p < 0.001$), respectively. Next, a multiple regression with both surprise and upvotes as predictors, produced significant coefficients for both upvotes (0.38, $z = 5.11, p < 0.001$) and surprise (0.12, $z = 6.28, p < 0.001$). We found similar results for a multiple regression with both social utility and upvotes as predictors, with both upvotes (0.16, $z = 2.59, p = 0.01$) and social utility (0.62, $z = 36.10, p < 0.001$) producing significant coefficients. We obtained similar results for a multiple regression with both usefulness and upvotes as predictors, with both upvotes (0.24, $z = 4.03, p < 0.001$) and usefulness (0.65, $z = 41.89, p < 0.001$) producing significant coefficients. This suggests that none of the judgements fully mediated the effect of upvotes on curiosity. We

Judgment	Coef	z	p-value
curiosity*	0.32	12.10	$< .001$
popularity*	0.08	3.51	$< .001$
confidence*	−0.09	−3.90	$< .001$
surprise*	−0.10	−4.32	$< .001$
social utility	0.00	0.03	$= 0.98$
usefulness*	0.11	3.22	$< .001$

Table S6

Multiple regression using curiosity, popularity, confidence, surprise, social-utility, and usefulness to predict whether a question was revealed (Experiment 2a); significant differences are starred.

further ran Sobel tests to confirm that usefulness ($z = 6.38, p < .001$), surprise ($z = 4.76, p < .001$), and social-utility ($z = 6.38, p < .001$) indeed partially mediated the effect of upvotes on curiosity.

Phase 2. Table S6 reports the results from the multiple regression using all six judgments to predict whether a question was revealed. We see that curiosity outperformed all other predictors with a coefficient of 0.32 ($z = 11.76, p < .001$).

Post-Number Condition

To further ensure that the effects of upvotes were not driven merely by their numerical content, we also performed a repeated measures ANOVA, with low and high upvote as a within-subjects comparison, condition (post-number vs. popularity) as a between subjects comparison, and curiosity as the dependent variable. We found that there was a significant interaction, $F(1,456) = 21.85, p < 0.001$, with the effect size (partial eta-square) equal to 0.05. We also performed this same analysis with usefulness, social-utility, and confidence as the dependent variables and found a significant interaction for usefulness, $F(1,456) = 9.22, p < 0.001$, and social utility, $F(1,456) = 28.66, p < 0.001$, but not for confidence, $F(1,456) = 1.43, p = 0.23$.

Baseline Condition

We compared ratings from the post-number and baseline conditions and found no ratings were significantly different from each other (curiosity: $t(293) = 0.81, p = 0.42$, confidence: $t(293) = 0.63, p = 0.53$, social utility: $t(293) = 0.67, p = 0.51$, usefulness: $t(293) = 0.06, p = 0.95$).

We then compared the average ratings given to low upvote and high upvote questions to the control ratings (i.e. the combined ratings for the post-number and baseline conditions). Table S7 reports the results. We see that mean curiosity, usefulness, and social-utility ratings for the low upvote questions were significantly lower points than that in the control condition, while the ratings for high upvote questions were not significantly different.

Judgment	Control rating compared to	t	*p*-value
curiosity	low-upvotes*	-3.79	< 0.01
	high-upvotes	-0.91	= 0.36
confidence	low-upvotes	0.08	= 0.93
	high-upvotes	-0.59	= 0.56
social utility	low-upvotes*	-3.18	< 0.01
	high-upvotes	-1.89	= 0.06
usefulness	low-upvotes*	-2.34	< 0.05
	high-upvotes	-1.22	= 0.23

Table S7

Comparison of the average ratings given to low upvote and high upvote questions to the control ratings (Experiment 2); significant differences are starred. We see that mean curiosity, usefulness, and social-utility ratings for the low upvote questions were significantly lower than that in the control condition, while the ratings for high upvote questions were not significantly different.

Experiment 2c: Replication of Experiment 2 up-vote condition

Method

Participants. 301 participants were recruited from Amazon Mechanical Turk and paid \$1.50 for their participation in a 12-minute study. Informed consent was obtained using a consent form approved by the Institutional Review Board at the University of California, Berkeley.

Stimuli. The stimuli used in this experiment were the same fifty questions used in Experiment 2 from the main paper.

Procedure. This experiment followed the same design and procedure as Experiment 2. However, we only had one condition in this experiment: the *upvote* condition. Thus, all participants were displayed the upvotes the questions received along with the question text. The goal of this experiment was to replicate the main findings of our paper, i.e., to test whether popularity influences curiosity and if that in turn influences the information participants choose to reveal.

Results

Phase 1. We first tested whether upvotes again succeeded in manipulating perceived popularity. As shown in Figure S2(a), perceived popularity was 2.20 points higher for high upvote questions when compared to low upvote questions. This was a significant difference, $t(300) = -22.3, p < .001$, suggesting that upvotes once again served as an effective social cue.

Next, we tested whether curiosity was affected by upvotes. As shown in Figure S2(a), curiosity was higher by 0.45 points for questions with high upvotes versus low upvotes and this difference was significant, $t(300) = -6.7, p < .001$. We also conducted similar paired-samples t-tests for each of the remaining judgments (refer to Table S8). We again found that popularity did not reliably affect participants' confidence, but it did have a significant effect on participants' judgments of surprise, social utility, and usefulness. Again, these judgments were all correlated with curiosity; but none fully mediated the effect of popularity on curiosity.

Phase 2. To investigate whether the manipulation of upvotes affected information search, we once again tested whether questions with high upvotes were revealed more often than the

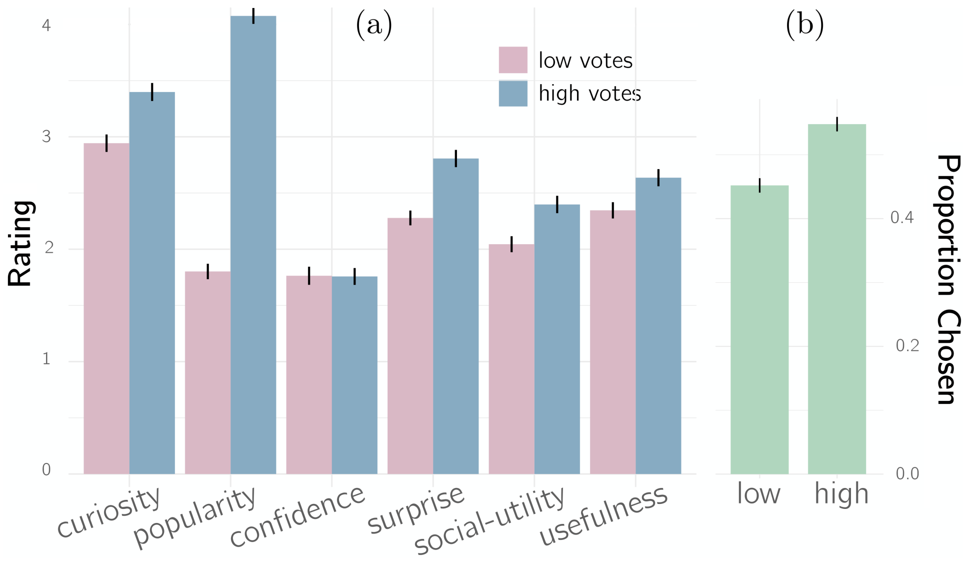

Figure S2. **Experiment 2c**. (a) Upvotes influenced participants' ratings for every judgment except for their confidence. (b) Higher upvoted questions were once again more likely to be revealed by participants.

Judgment	Difference Score	$t(300)$	p-value
popularity*	2.20	−22.3	< 0.001
curiosity*	0.45	−6.7	< 0.001
confidence	−0.001	0.06	0.95
surprise*	0.51	−6.45	< 0.001
social utility*	0.34	−6.37	< 0.001
usefulness*	0.28	−4.85	< 0.001

Table S8

Impact of manipulating upvotes on judgment ratings. *Difference Scores are the mean difference of ratings, subtracting the mean ratings for low upvote questions from those for high upvote questions; significant differences are starred.*

chance value of 50% (Refer to Figure S2(b)). A single-sample t-test showed that participants' choice of high upvote questions (54.8% of the time) was significantly different from chance,

$t(300) = 4.25, p < .001$. This demonstrates that manipulating upvotes once again had an effect on information search.

Lastly, we tested whether the effect of upvotes on information search was mediated by curiosity. First, a logistic regression predicting question choice from the manipulation of upvotes revealed a significant positive coefficient of 0.38 ($z = 5.21, p < .001$). A similar regression with curiosity as the predictor produced a coefficient of 0.40 ($z = 18.68, p < .001$). Next, a multiple regression with both curiosity and upvote level resulted in a coefficient of 0.25 for upvotes ($z = 3.2, p = .001$) and curiosity remained significant at 0.39 ($z = 18.33, p < .001$). This suggests that the effect of upvotes on information search was mediated by curiosity. Finally, we considered whether the effect of curiosity was still significant, controlling for all other judgments. We conducted a multiple regression using all six judgments to predict whether a question was revealed and found that curiosity outperformed all other predictors with a coefficient of 0.34, maintaining its significance ($z = 12.74, p < .001$).

This experiment replicates our findings from the upvote condition of Experiment 2 and demonstrates that popularity influences curiosity, which in turn influences information search.

Additional analyses

Relationship between curiosity and confidence

A large number of studies have found an inverted U-shaped relationship between curiosity and confidence (Kang et al., 2009; Baranes et al., 2015). In essence, these studies have shown that people's curiosity is highest for stimuli for which they have a moderate level of confidence. We analyzed data for both Experiment 2 and 2b, to study whether such an effect exists in our data as well (note that since we didn't ask for confidence ratings in Experiment 1, we don't perform this analysis for Experiment 1). Figure S3 plots the normalized mean curiosity rating with respect to transformed confidence (0–1) for all conditions in both Exp 2 and 2b. From the figures, we see that an inverted U-shaped relationship exists between curiosity and confidence, i.e., curiosity peaks at intermediate levels but becomes lower for the lowest or the highest levels of confidence for all the

conditions in both Exp 2 and 2b. Following the method of previous papers (Kang et al., 2009; Baranes et al., 2015), we fitted the data to the equation: curiosity = $b_0 + b_1 \times c + b_2 \times c \times (1-c)$ to all conditions in Exp 2 and 2b, where c was the re-scaled confidence score. For the upvote condition in Exp 2, the model provided r = 0.14 and a significant coefficient for the quadratic coefficient i.e. c × (1-c) (b_2 estimate = 2.13, $p < 0.05$). Similarly, for the baseline condition in Exp 2 (after combining data from the post-number and baseline conditions), the model provided r = 0.1 and a significant coefficient for the quadratic coefficient (b_2 estimate = 1.5, $p < 0.05$). Similar results also followed for Exp 2b. For the upvote condition in Exp 2b, the model provided r = 0.15 and a significant coefficient for the quadratic coefficient (b_2 estimate = 2.1, $p < 0.05$). For the baseline condition in Exp 2b, the model provided r = 0.14 and a significant coefficient for the quadratic coefficient (b_2 estimate = 2.5, $p < 0.05$). In this way, our results also serve as a replication of previous empirical findings on curiosity, albeit on a different set of stimuli, namely everyday scientific questions.

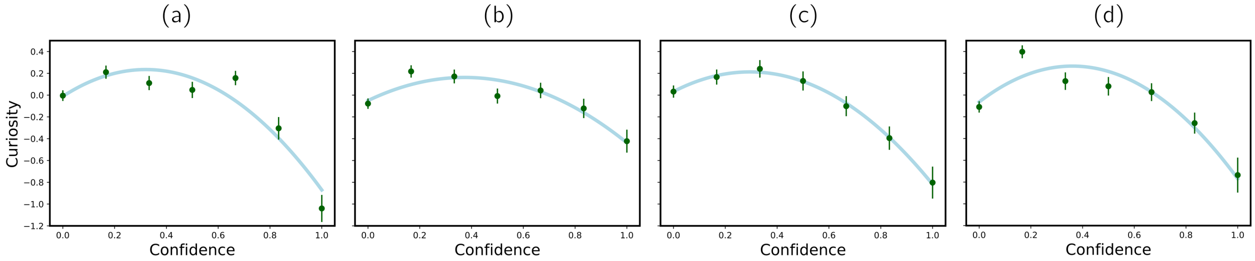

Figure S3. **Relationship between curiosity and confidence**. (a) Upvote condition in Exp 2 (b) Baseline condition in Exp 2 (after combining data from the post-number and baseline conditions) (c) Upvote condition in Exp 2b (d) Baseline condition in Exp 2b. We note that an inverted U-shape relationship exists between curiosity and confidence for all the conditions.

References

Baranes, A., Oudeyer, P.-Y., & Gottlieb, J. (2015). Eye movements reveal epistemic curiosity in human observers. *Vision research*, *117*, 81–90.

Kang, M. J., Hsu, M., Krajbich, I. M., Loewenstein, G., McClure, S. M., Wang, J. T.-Y., & Camerer, C. F. (2009). The wick in the candle of learning: Epistemic curiosity activates reward circuitry and enhances memory. *Psychological Science*, *20*(8), 963–973.
